# Delirium screening and alerting systems for older hospital inpatients

**DOI:** 10.1186/s12913-025-12829-z

**Published:** 2025-05-07

**Authors:** Lakeshia Benn, Nirav Shah, Amy McKinney, Lillian Min, Ilyas Aleem, Matthew Luzum, Phillip E. Vlisides

**Affiliations:** 1 Department of Inpatient Rehabilitation, Michigan Medicine, Ann Arbor, MI USA; 2 College of Health Professions & McAuley School of Nursing, University of Detroit Mercy, Detroit, MI USA; 3 Department of Anesthesiology, University of Michigan Medical School, 1H247 UH, SPC-5048, 1500 East Medical Center Drive, MI 48109-5048 Ann Arbor, USA; 4 Department of Internal Medicine, Division of Geriatric & Palliative Medicine, Michigan Medicine, Ann Arbor, MI USA; 5 VA Ann Arbor Healthcare System, Department of Internal Medicine, Division of Geriatric Research, Education, and Clinical Center (GRECC), Ann Arbor, MI USA; 6 Department of Orthopaedic Surgery, Michigan Medicine, Ann Arbor, MI USA; 7 Department of Internal Medicine, Michigan Medicine, Ann Arbor, MI USA; 8 Center for Consciousness Science, University of Michigan Medical School, Ann Arbor, MI USA

**Keywords:** Decision support (clinical), Delirium, Evaluation methodology, Implementation science, Quality improvement

## Abstract

**Background:**

Delirium often goes unrecognized in the hospital, leading to missed opportunities for management. The objective of this study was to test a multicomponent program for delirium screening and reporting for older, hospitalized adults.

**Methods:**

We implemented a multicomponent delirium screening and alerting program within two university hospital units for all patients ≥ 70 years of age. The initiative compared performance of the 4 ‘A’s Test, Nursing Delirium Screening Scale, and Confusion Assessment Method. Additionally, the study team provided recurrent educational sessions with nurses and implemented pager and electronic health record alerts for patients who screened positive for delirium. Nurses were then surveyed about their perspectives, and clinical outcomes were abstracted from the medical record.

**Results:**

Compared to the Confusion Assessment Method, the proportion of positive screens was significantly higher (positive screens/admissions) with the 4 ‘A’s Test (49/448, 11% vs. 12/399, 3%, *p* < 0.001) and the Nursing Delirium Screening Scale (83/539, 15% vs. 12/399, 3%, *p* < 0.001). Among surveyed nurses, 32/41 (78%) expressed that the alerting system provided at least “moderate” motivation to screen for delirium, and 35/41 (85%) voiced that it provided at least “moderate” motivation to record positive screens. Most respondents (23/42, 55%) reported recurrent educational sessions as “very helpful.” Positive screens were associated with higher mortality (6.6% vs. 1.9%, *p* = 0.003), longer hospitalizations (13 [± 11] days vs. 7 [± 11], *p* < 0.001), and higher likelihood of discharge to care facilities (45% vs. 23%, *p* < 0.001).

**Conclusions:**

Positive delirium screening rates were higher with the 4AT and NuDesc compared to the CAM. Additionally, alerting systems and educational initiatives served as motivating factors for delirium screening and charting.

**Supplementary Information:**

The online version contains supplementary material available at 10.1186/s12913-025-12829-z.

## Introduction

Delirium reflects an acute change in attention and related cognitive functions that affects 20–50% of older hospitalized patients [1, 2]. When delirium occurs, the experience is often distressing for both patients and families, and the syndrome may persist after hospital discharge [3]. Moreover, delirium is associated with prolonged hospitalization [4], future cognitive and functional decline [5, 6], and increased mortality [7]. To successfully manage delirium, a fundamental step is to consistently identify – and report – positive delirium screens. Unfortunately, delirium is often underrecognized and underreported, in part due to distinct challenges that arise with delirium screening [8–10]. By extension, delirium management opportunities are often missed given that delirium is inconsistently identified on hospital inpatient units.

Based on an internal audit at our institution, we found that positive delirium screens (via Confusion Assessment Method) were only charted for approximately 1% of all inpatient adults on a major, representative inpatient surgical unit and 5% on a corresponding medical unit. However, previous observational studies and trials involving these units have revealed an approximate 20% delirium incidence when assessed via trained delirium research team [2, 11]. Previous quality improvement studies have identified barriers to delirium screening, which often include inadequate training with screening tools, demanding clinical workload, and an institutional culture that does not prioritize delirium screening [8, 9]. These challenges likely contribute to the low sensitivity (~ 30%) of delirium screening tools encountered the routine clinical setting [12]. Successful delirium screening is a foundational component to a delirium quality improvement program, as subsequent evaluation and management steps cannot occur until delirium is first identified.

The objective of this study was thus to test a multicomponent program for improving delirium screening and reporting in older patients (≥ 70 years) on two typical inpatient units in a major university hospital setting. The approach to achieving this objective was to perform a quality improvement initiative that compared different nursing-based validated delirium screening tools, incorporated recurrent delirium education sessions, and tested positive delirium screen alerting systems for improving the clinical environment surrounding delirium care.

## Methods

### Study design and overview

This was a quality improvement initiative at a major tertiary care center (Michigan Medicine, Ann Arbor, MI USA) and was granted exemption from the University of Michigan Medical School Institutional Review Board (HUM00228111). The initiative took place over 10 months (April 2023 – February 2024) on two inpatient units – one medical, one surgical – with low positive delirium screening rates. All nurses who provide direct patient care on these units were eligible to participate. As a pragmatic initiative, all patients ≥ 70 years of age admitted as an inpatient to these units were included and eligible for analysis. This study was also conducted in accordance with the Standards for Quality Improvement Reporting Excellence Checklist (2.0) [13]. 

### Quality improvement initiatives and interventions

**Fig. 1:**
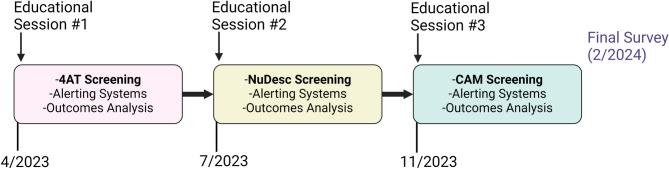
Overall study flow presented. Educational sessions were provided at the beginning of each block, which included background education on delirium and training specific to each subsequent delirium screening tool in use for a given period. 4AT, 4 ‘A’s Test; NuDesc, Nursing Delirium Screening Scale; CAM, Confusion Assessment Method

The initiative tested multiple components for improving delirium screening, charting, and management. First, given the low delirium detection rates at our institution with the Confusion Assessment Method (CAM), the 4 ‘A’s Rapid Clinical Test for Delirium (4AT) and Nursing Delirium Screening Scale (NuDesc) were tested as possible alternatives to the CAM [14–16] (Table 1). Each instrument was used by bedside nurses for a period of approximately three months in sequential order (4AT, NuDesc, then CAM) (Fig. 1). These screening tools have been validated and demonstrate similar sensitivities and specificities when administered by clinicians in the inpatient setting [17–22]. A score of 4 or higher on the 4AT was the threshold for a positive screen, and a score of 2 or higher was used for the NuDesc [14, 15]. Nurses were assigned to complete delirium screens at least once per day shift (7:00 AM – 7:00 PM) and again during the night shift (7:00 PM – 7:00 AM), which reflects standard practice at our institution.

**Table 1:** Delirium screening tool comparisons

Screening Tool	Description
4 ‘A’s test (4AT)	Four cognitive domains are rapidly assessed in the clinical setting: alertness, orientation, attention, and acute change/fluctuating course. A final score is provided, with scores ≥ 4 points indicating possible delirium (± cognitive impairment) and scores 1–3 indicating possible cognitive impairment. Pooled sensitivity and specificity across various clinical settings are each approximately 88% [14, 18].
Nursing Delirium Screening Scale (NuDesc)	This is a tool designed for nurses to complete at the end of each shift, drawing from their experiences throughout the shift. The screening tool includes assessment of five relevant areas of cognition and arousal: disorientation, inappropriate behaviour, inappropriate communication, illusions/hallucinations, and psychomotor retardation. A continuous score is provided, but a threshold (≥ 2) suggests delirium presence. Sensitivity approximately 86%, specificity 87% [15].
Confusion Assessment Method (CAM)	The Confusion Assessment Method (CAM) conventionally consists of a formal cognitive function assessment paired with subsequent diagnostic algorithm for determining delirium. Multiple cognitive domains are assessed, and the final diagnostic algorithm is based on acute change, fluctuating course, inattention, disorganized thinking, and altered level of consciousness. Sensitivity from pooled high-quality studies is approximately 82% with specificity of 99% [19]. Using the CAM based on bedside observations alone, without cognitive function testing, renders a lower sensitivity (19 − 67%) but a preserved specificity (91–98%) [20, 21].

Second, a Clinical Nurse Specialist with delirium expertise (LB) led educational delirium sessions with unit nurses approximately every three months, right before introduction of the next sequential delirium screening tool (Fig. 1). These educational sessions also included training on each specific screening tool. Pre- and post-educational session tests were also conducted to assess knowledge gains. The Clinical Nurse Specialist also conducted rounds to monitor progress of the initiative, address questions, and provide further support to unit nursing as needed.

Lastly, an electronic alerting system was then tested when a positive delirium screen was recorded by a nurse in the electronic health record. Specifically, upon recording the positive screen, a pager alert was sent to the primary team clinician (e.g., physician, nurse practitioner), charge nurse, and pharmacist notifying them of the positive delirium screen. An additional alert would also populate in the electronic health record alert upon chart opening (Supplementary Text S1). Both alerts would provide recommendations and refer clinicians to a hospital delirium management order set.

### Data acquisition

Electronic health record reports were generated from Epic (Epic Systems, Madison WI, USA) to determine the number of admissions for each period (4AT, 4/20/2023–7/23/2023; NuDesc, 7/24/2023–11/12/2023; CAM, 11/13/2023–2/17/2024). Report filters were set to only include patients ≥ 70 years of age admitted and discharged from the designated units during the initiative time periods. The study team held an alert pager and manually logged all positive delirium alert patients during the initiative. Then, patients for whom an alert was sent were manually reviewed in the medical record by the study team to (1) confirm the positive delirium screen and (2) verify the delirium screen resulted from the correct delirium tool (e.g., 4AT, NuDesc, CAM) being used for the designated time period. These strategies resulted in the final numerator, denominator, and proportions (%) in the Results section pertaining to delirium screening. Survey data (see Outcomes section below) were collected directly from participating nurses.

### Outcomes

The incidence of positive screens (i.e., any occurrence of a positive screen during inpatient hospitalization) was recorded via electronic health record reports. This was the primary outcome and focus of the current initiative, given the low positive screening rates currently charted at our institution. Screening tool adherence was also tracked, and this was calculated by determining the proportion of patients who had at least one screen completed during their inpatient stay. Positive screen incidence and adherence were then compared among the three tools.

At the end of the initiative, a survey was distributed to participating nurses to gain perspectives on various aspects of the quality improvement initiative, including the different delirium screening tools tested and alerting system value. To develop nursing surveys, an iterative process of instrument development was employed, using item generation and reduction via team workshops, which included the initiative leadership team, a hospital geriatrician, and nursing leadership representatives. The survey was then pilot tested with a small group of nurses prior for final edits and refinement. Nurses were then offered the choice to complete the survey via online platform (Qualtrics Survey Software, Provo, UT USA) or paper (Supplementary Text S2).

Lastly, four clinical outcomes were evaluated as a secondary analysis: (1) hospital mortality, (2) observed vs. expected length of stay based on Diagnosis-Related Group (DRG), (3) discharge disposition, and (4) utilization of services weighted by Relative Value Units (RVUs) summed across hospital days. The RVU-based measure was a summary of all procedures, including professional billing codes matched with RVUs. These outcomes were then compared between patients ≥ 70 years old admitted to the same units during the study timeframe with positive delirium screens (*n* = 152 hospitalizations) and those without a positive screen (*n* = 524 hospitalizations).

### Statistical analysis

Hospital mortality and discharge destination were evaluated by one-way analysis of variance. Length of stay (days) between groups was compared via a multivariable regression, controlling for age, sex, comorbidity (Charlson Comorbidity Index), and expected length of stay based on DRG for each hospitalization. RVUs were also compared via regression, controlling for age, sex, comorbidity, and adjusted length of stay (observed length of stay divided by expected length of stay). The model was an exponential means regression that predicted outcomes and confidence intervals of skewed outcomes, such as length of stay and RVUs. The model also included a random effect for hospital admissions nested within patients. Complete case counts were used for all lines of analysis; no imputation procedures were performed. Lastly, there was no planned a priori sample size with respect to patient volume or survey analysis. Final sample sizes reflected convenience sampling based on all available patients and nurses, respectively. All analyses were conducted with Stata 18.5 (Copyright © 1985–2023 by StataCorp LLC, College Station, TX, USA) and SPSS (IBM SPSS Statistics version 24.0 for Windows, IBM Corp. Armonk, NY USA).

## Results

The quality improvement initiative took place from 4/20/2023–2/17/2024, with each screening tool implemented for a three-month block (Fig. 1). The incidence of positive delirium screens was highest with the 4AT and NuDesc, and incidence of positive screens with the CAM was similar to historical controls (Table 2). Conversely, screening adherence was significantly higher with the CAM compared to the two other instruments (Table 2).

**Table 2:** Delirium screening and adherence results

Screening Tool	Positive screens (*n*)/Admissions (*n*)	Positive screen incidence (%)
4AT	49/448	11% (*p* < 0.001)*
NuDesc	83/539	15% (*p* < 0.001)*
CAM	12/399	3%*
Historical CAM (2021) -Inpatient surgical unit	--	1.2%
Historical CAM (2021) -Inpatient medical unit	--	4.7%
Screening Tool	Screened patients (n)^†^/Admissions (n)	Screening adherence, (%)
4AT	404/448	90% (*p* < 0.001)*
NuDesc	452/583	78% (*p* < 0.001)*
CAM	396/399	99%*

*Statistical comparisons via chi-squared testing comparing 4AT to the CAM and NuDesc to the CAM. The 4AT was implemented from 4/20/2023–7/23/2023, NuDesc from 7/24/2023–11/12/2023, and CAM from 11/13/2023–2/17/2024. Precise numerator and denominator data unavailable for historical CAM data, which were derived from the 2021 calendar year

^†^Patients who had at least one delirium screen performed during admission

Participating nurses (*n* = 46/130, 35% response rate) then completed a survey to express their experiences and perceptions related to delirium and the initiative. Median (interquartile range) age was 34 (27–40) years old, 33/43 respondents (77%) identified as female, with a median of 4 (2.5–7) years working on an acute care inpatient unit (additional data available in Supplementary Text S3). All respondents expressed that recurrent, structured educational sessions were at least “somewhat helpful,” and the majority of respondents (23/42, 55%) reported the sessions as “very helpful.” Nurses also expressed an ongoing desire to incorporate pager alerts in the clinical workflow that would alert covering clinician teams to a positive delirium screen. In total, 32/41 (78%) respondents expressed that pager alerts provide at least “moderate” motivation to screen for delirium, and 35/41 (85%) voiced that these alerts provided at least “moderate” motivation to record positive screens in the electronic health record (Supplementary Text S3). Lastly, none of the delirium instruments tested were perceived as time-consuming or difficult to use; the top choice was the CAM (17/38, 45%), followed by the NuDesc (15/38, 39%), then 4AT (6/38, 16%). Complete results are included in Supplementary Text S3.

Patients who screened positive for delirium, based on all three instruments, were significantly older (mean [± standard deviation] age 82 [± 7] years compared to 78 [± 6], *p* < 0.001); stayed in the hospital for a longer period of time (mean 13 [± 11] days vs. 7 [± 11], *p* < 0.001); were more likely to be discharged to skilled care facilities (45% vs. 23%, *p* < 0.001); and demonstrated higher wRVU usage (66 [± 57] vs. 48 [± 47] wRVUs, *p* < 0.001) (Cohort characteristics presented in Supplementary Table S4). After controlling for age, sex, comorbidity burden, and expected length of stay, screening positive for delirium was still associated with 14.1 extra wRVUs (95% CI 10.9 to 17.3 extra RVUs, *p* < 0.0001). Additionally, after adjusting for the same confounders, a positive delirium screen was still associated with an increased hospital length of stay (6 [95% CI: 5–7] additional days, *p* < 0.0001). Mortality was also higher in patients who screened positive for delirium (6.6% vs. 1.9%, respectively, *p* = 0.003). Finally, based on DRG data, patients who screened positive for delirium were more likely to be admitted for infectious and neurological conditions; patients who were admitted for orthopedic and gastrointestinal reasons were less likely to screen positive for delirium (Supplementary Text S5).

## Discussion

This quality improvement initiative revealed that positive delirium screening rates vary based on the screening tool used, with the 4AT and NuDesc demonstrating the highest incidence of positive delirium screens. Patients screening positive for delirium demonstrated increased mortality, higher likelihood of discharge to skilled care facilities, and increased healthcare utilization, affirming that nursing-based delirium screens can be successfully used for identifying patients with high cognitive and clinical vulnerability. Survey analysis showed varying preferences with respect to delirium screening tool, but most nurses voiced that pager and health record alerts served as motivating tools for recording positive delirium screens.

One of the most striking findings was the variance in positive delirium screen incidence across the instruments tested. While the CAM is the most widely studied and has been extensively validated [19, 23], correct usage is incumbent upon formal cognitive function testing prior to CAM scoring, and diagnostic accuracy is reduced when based on bedside observations alone without formal cognitive function testing [20, 21]. This may explain in part why screening incidence was lowest with the CAM in this initiative, as nurses from these units previously expressed lack of standardized training and cognitive function testing with the CAM [10]. Moreover, incorporating formal cognitive function testing within a busy clinical workflow prior to CAM assessments may be impractical given the demands of clinical care [24]. By comparison, the 4AT does not require formal training or a preceding cognitive function test, and both the sensitivity and specificity remain comparable to the CAM [14, 17]. In mixed hospital inpatient settings, including older patients and those with dementia, the 4AT also demonstrates high sensitivity and specificity compared to other tools [17]. However, the uptake and acceptability may ultimately depend on ease of integration within clinical workflow. For example, the NuDesc requires a 24-hour observation cycle across multiple nursing shifts and may thus not be appropriate for short-stay units. Screening adherence was highest with the CAM, which may reflect institutional experience and pre-established workflow integration. Ultimately, the optimal delirium screening tool for a given setting will thus depend on multiple factors, including acceptability by clinicians performing the screens and whether the instrument characteristics are appropriate for a given hospital unit, based on staffing patterns and patient characteristics.

It is also important to note that, with the positive delirium screens charted in this initiative, nurses were able to independently identify vulnerable patients who were more likely to require additional healthcare resources, discharge to skilled care facilities, or die during hospitalization, all during routine clinical workflow. No additional oversight, extensive training, or incorporation of additional cognitive function testing were required. Implementing a clinically pragmatic delirium screening program, which includes routine documentation of positive screens, is important for maintaining an electronic health record database that can be used for delirium quality assurance and research programs. Most positive screens charted were generated from the 4AT and NuDesc, suggesting that these relatively brief instruments perform well with identifying high-risk patients and can be considered as alternatives to more involved and potentially time-consuming strategies that require dedicated training, such as cognitive function testing paired with the full CAM algorithm. In fact, the incidence of positive CAM screens in the current study was similar to historical controls (Table 2), despite the recurrent educational sessions and training provided with this initiative. This suggests that there may be additional barriers for sufficiently identifying and recording positive CAM screens.

Lastly, institutional environment plays a critical role in supporting best practices for delirium prevention and management, and delirium education should be a central component of organizational culture. Indeed, nurses in this program have previously cited the need for structured education pertaining to delirium, including screening instrument training [10]. In response, delirium educational sessions were provided to unit nurses every three months for this initiative, including dedicated training on screening forms. The majority of nurses surveyed found these sessions helpful and reported that they should continue on a biannual or triannual basis. Indeed, for maintaining sustainability of successful delirium management programs, standing educational and training sessions – with a clinician specialist – is likely required. In addition to recurrent education, nurses also voiced the desire for more robust communication pathways with physicians and other clinicians with positive delirium screens. Alerts were thus incorporated into our paging and electronic medical record system, as previously described. Nurses expressed that this alerting system served as a motivating factor to screen for delirium and record positive screens (Supplementary Text S3). Sustained delirium communication and care pathways may help maintain consistent charting and reporting of positive screens, as evidenced by this current initiative. While this quality initiative did not test the direct impact of these alerts on clinical outcomes, this may be worth testing in future, follow-up studies, particularly given that the alerts can accelerate clinician response pathways [25], and they served a motivating factor to identify delirium in this current initiative.

Important study limitations are worth noting. This was a small quality improvement initiative restricted to two inpatient units. Findings from the study, including the incidence of delirium screens from each instrument tested, may have varied with the inclusion of additional hospital units and patient characteristics. The individual total number of screens was not tracked, but the proportion of patients with at least one screen was reported via electronic health record reporting system (Table 2), and this is the metric made available for tracking at our hospital. Additionally, while validated screening tools were tested, no concurrent gold standard assessment (e.g., via DSM criteria by a trained clinician) was conducted in this study to verify delirium diagnosis for each patient. While screening characteristics (e.g., positive rates) could thus be analyzed, assessment accuracy was not tested in this study. It also is noteworthy that clinicians can identify vulnerable, older patients via signs of frailty, observable both at the bedside and via chart review [26, 27]. Frailty screening may serve as a complementary strategy to hospital delirium screening for older adults. Because the survey response rate was relatively low, the breadth of nursing perspectives and experiences captured was limited. Lastly, as this initiative was restricted primarily to nurses, perspectives from other clinicians (e.g., physicians) were not included.

## Conclusions

In summary, hospital delirium screening and charting may be improved by recurrent educational sessions, implementation of delirium alerting systems, and using a delirium screening tool conducive to the characteristics of a given hospital unit.

## Supplementary Information

Supplementary Material 1.

## Data Availability

The datasets used and/or analyzed during the current study are available from the corresponding author on reasonable request.
