# Supplementary material for: Delirium screening and alerting systems for older hospital inpatients
Source: BMC Health Serv Res. 2025 May 7;25:655. doi: 10.1186/s12913-025-12829-z (PMC12060402; doi:10.1186/s12913-025-12829-z)
Supplement: Supplementary file 1 — Supplementary Material 1. [file 12913_2025_12829_MOESM1_ESM.docx]

**Delirium Screening and Alerting Systems for Older Hospital Inpatients**

**Supplementary Materials**

Table of Contents

[**I.** **Supplementary Text S1 – Positive Delirium Screen Alert** 2](#_Toc195692292)

[**II.** **Supplementary Text S2 – Nursing Survey** 2](#_Toc195692293)

[**III.** **Supplementary Text S3 – Full Survey Results (n=46 total respondents)** 15](#_Toc195692294)

[**IV.** **Supplementary Table S4 – Cohort Characteristics** 46](#_Toc195692295)

[**V.** **Supplementary Text S5 – Admissions Based on Diagnosis-Related Group** 48](#_Toc195692296)

# **Supplementary Text S1 – Positive Delirium Screen Alert**

The following alert would populate in the electronic medical record patient chart when a positive delirium screen was recorded:


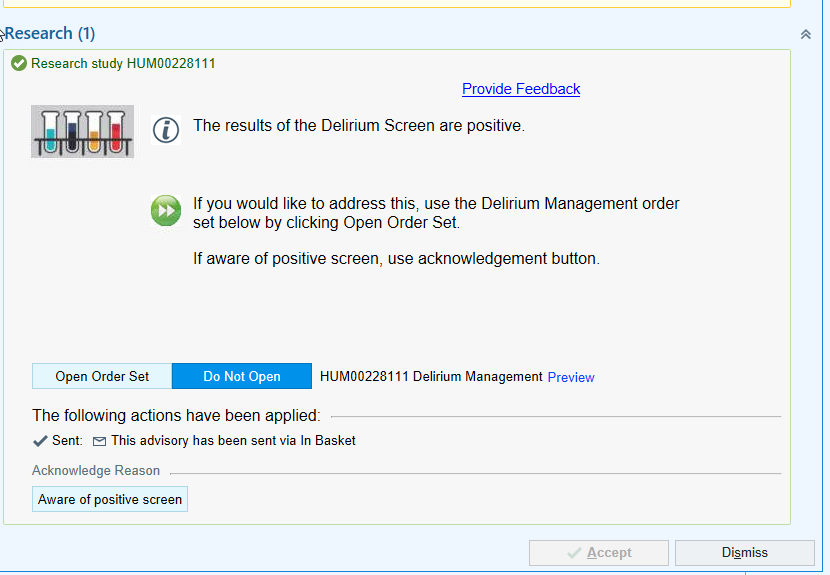


And the pager alert would read as follows: “Patient (NAME, MRN, location) has screened positive for delirium. Please evaluate the patient, review medications and laboratory/study results, and consider the MiChart delirium management order set.”

# **Supplementary Text S2 – Nursing Survey**

Final Nursing Delirium Survey - UM CQI

Start of Block: Instructions

**Instructions:**Please only complete this survey once. If you have already completed this online, do not complete this survey on paper. Likewise, if you have completed this on paper already, do not complete this online.

End of Block: Instructions

Start of Block: Background Questions

Q1 What is your current age?

________________________________________________________________

Q2 What gender do you identify with?

- Male (1)
- Female (2)
- Non-binary / third gender (3)
- Prefer not to say (4)

Q3 What is your race?

- White (1)
- Black or African American (2)
- Asian (3)
- Pacific Islander (4)
- Native American (5)
- Other (6)
- Prefer not to respond (7)

Q4 What is your ethnicity?

- Hispanic (1)
- Non-Hispanic (2)

Q5 What is the total number of years you have worked on a non-ICU inpatient hospital unit (such as your current one)?

________________________________________________________________

Q6 What is the total number of days per week you provide in-person bedside care?

________________________________________________________________

Q10 Please estimate the percentage of patients that you care for on a weekly basis who are at least 70 years old:

- 0-25% (1)
- 26-50% (2)
- 51-75% (3)
- 76-100% (4)

End of Block: Background Questions

Start of Block: Delirium Education

Q7 Recurrent delirium educational sessions have been occurring over the past year. How helpful have these sessions been pertaining to delirium care?

- Not at all helpful (1)
- Not so helpful (2)
- Somewhat helpful (3)
- Very helpful (4)
- Extremely helpful (5)

Q9 What is the optimal frequency of delirium educational sessions?

- Once yearly (1)
- Twice yearly (2)
- Every 3-4 months (3)
- Monthly (4)
- Weekly (5)

Q8 Additional comments on delirium education:

________________________________________________________________

End of Block: Delirium Education

Start of Block: Delirium Charting and Alerting

Q11 During these past several months, when nurses on your unit recorded a positive delirium screen in MiChart, this would send alerts (pager and Best Practice Alert) to the covering physician, NP, or PA. Were you aware of this?

- Yes (1)
- No (2)

Display This Question:

If Q11 = Yes

Q12 If yes, how much did this information (i.e., that a pager alert and Best Practice Alert would be sent to the covering clinician) motivate you to screen for delirium?

- Not at all (1)
- Slightly (2)
- Moderately (3)
- Very much (4)
- Extremely (5)

| Page Break |  |
| --- | --- |

Display This Question:

If Q11 = Yes

Q13 If yes, how much did this information (i.e., that a pager alert and Best Practice Alert would be sent to the covering clinician) motivate you to record positive delirium screens?

- Not at all (1)
- Slightly (2)
- Moderately (3)
- Very much (4)
- Extremely (5)

Q14 Moving forward, how much would this information (i.e., that a pager alert and/or Best Practice Alert would be sent to the covering clinician with a positive delirium nursing screen) motivate you to screen for delirium?

- Not at all (1)
- Slightly (2)
- Moderately (3)
- Very much (4)
- Extremely (5)

Q15 Moving forward, how much would this information (i.e., that a pager alert and/or Best Practice Alert would be sent to the covering clinician with a positive delirium nursing screen) motivate you to record positive delirium screens?

- Not at all (1)
- Slightly (2)
- Moderately (3)
- Very much (4)
- Extremely (5)

End of Block: Delirium Charting and Alerting

Start of Block: Delirium Screen Tools

Q16 Was the 4AT time-consuming?

- Not at all (1)
- Slightly (2)
- Moderately (3)
- Very much (4)
- Extremely (5)

Q17 Was the NuDesc time-consuming?

- Not at all (1)
- Slightly (2)
- Moderately (3)
- Very much (4)
- Extremely (5)

Q18 Was the CAM time-consuming?

- Not at all (1)
- Slightly (2)
- Moderately (3)
- Very much (4)
- Extremely (5)

Q19 How user-friendly was the 4AT?

- Very difficult (1)
- Difficult (2)
- Neutral (3)
- Easy (4)
- Very easy (5)

Q20 How user-friendly was the NuDesc?

- Very difficult (1)
- Difficult (2)
- Neutral (3)
- Easy (4)
- Very easy (5)

Q21 How user-friendly was the CAM?

- Very difficult (1)
- Difficult (2)
- Neutral (3)
- Easy (4)
- Very easy (5)

Q23 In your view, how accurate was the 4AT with identifying delirium?

- Poor (1)
- Fair (2)
- Good (3)
- Very good (4)
- Excellent (5)

Q24 In your view, how accurate was the NuDesc with identifying delirium?

- Poor (1)
- Fair (2)
- Good (3)
- Very good (4)
- Excellent (5)

Q25 In your view, how accurate was the CAM with identifying delirium?

- Poor (1)
- Fair (2)
- Good (3)
- Very good (4)
- Excellent (5)

Q26 What is your comfort level with the 4AT?

- Not at all comfortable (1)
- Slightly comfortable (2)
- Moderately comfortable (3)
- Very much comfortable (4)
- Extremely comfortable (5)

Q27 What is your comfort level with the NuDesc?

- Not at all comfortable (1)
- Slightly comfortable (2)
- Moderately comfortable (3)
- Very much comfortable (4)
- Extremely comfortable (5)

Q28 What is your comfort level with the CAM?

- Not at all comfortable (1)
- Slightly comfortable (2)
- Moderately comfortable (3)
- Very much comfortable (4)
- Extremely comfortable (5)

End of Block: Delirium Screen Tools

Start of Block: Final Questions

Q29 Please rank in order your preferred delirium screening tool (1 - most preferred, 2 - second most preferred, 3 - last preferred):

______ 4AT (April - July 2023) (1)

______ NuDesc (July - November 2023) (2)

______ CAM (November 2023 - February 2024) (3)

Q30 Please share any additional information that would be useful pertaining to delirium screening and management:

________________________________________________________________

Q31 Would you be willing to be contacted to discuss your answers further? If so, please write down your email address:

________________________________________________________________

End of Block: Final Questions

# **Supplementary Text S3 – Full Survey Results (n=46 total respondents)**

Q1. What is your current age?

42

44

29

40

34

30

37

28

30

25

25

27

26

33

25

26

31

44

25

34

40

53

51

35

35

38

29

39

27

25

43

25

23

52

32

30

34

40

36

40

**n=40 responses**

**Median (interquartile range) age: 34 (27 – 40)**

Q2. What gender do you identify with?

| Male | 9 |
| --- | --- |
| Female | 33 |
| Non-binary / third gender | 1 |
| Prefer not to say | 0 |
| Total | 43 |

Q3. What is your race?

| White | 35 |
| --- | --- |
| Black or African American | 2 |
| Asian | 2 |
| Pacific Islander | 1 |
| Native American | 0 |
| Other | 2 |
| Prefer not to respond | 1 |
| Total | 43 |

Q4. What is your ethnicity?


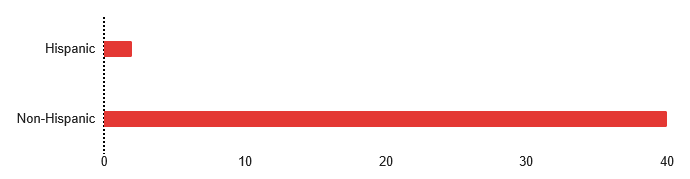


| Hispanic | 2 |
| --- | --- |
| Non-Hispanic | 40 |
| Total | 42 |

Q5. What is the total number of years you have worked on a non-ICU inpatient hospital unit (such as your current one)?

15

4.5

6

5

2

4

19

2

3

2+

3

6

1

5.5

5

1

8

7

12

3

18

2.5

2

4

2

3

1 1/2

3

3

8

3

1

1.5

3

3

8.5

2

4

4.5

19

5

12

**n=39 responses**

**Median (interquartile range) years: 4 (2.5 – 7)**

Q6. What is the total number of days per week you provide in-person bedside care?

2

4

3

2

3

3

3

3

3

5

3

2

3

32

3

3

2

3

3

3

2

3

3

3

3

3

3

3

2

5

3

4

3

4

3

4

3

2.5

4

2

4

4

3

**n=42 responses**

**Median (interquartile range) days: 3 (3 – 3)**

Q7. Please estimate the percentage of patients that you care for on a weekly basis who are at least 70 years old:

**
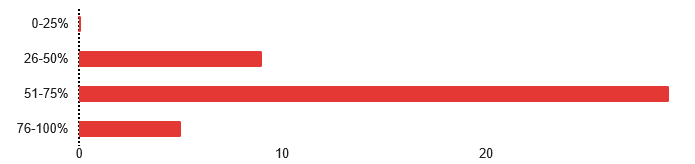
**

| 0-25% | 0 |
| --- | --- |
| 26-50% | 9 |
| 51-75% | 29 |
| 76-100% | 5 |
| Total | 43 |

Q8. Recurrent delirium educational sessions have been occurring over the past year. How helpful have these sessions been pertaining to delirium care?


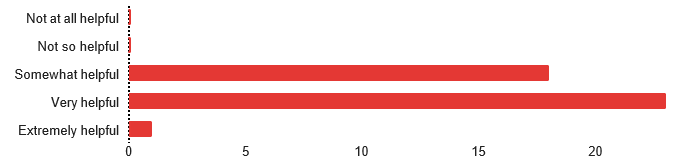


| Not at all helpful | 0 |
| --- | --- |
| Not so helpful | 0 |
| Somewhat helpful | 18 |
| Very helpful | 23 |
| Extremely helpful | 1 |
| Total | 42 |

Q9. What is the optimal frequency of delirium education sessions?


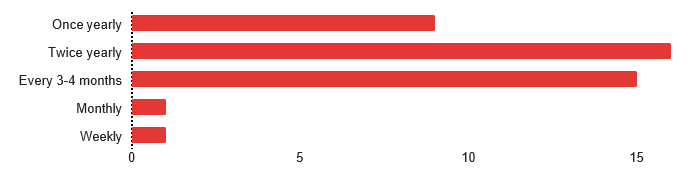


| Once yearly | 9 |
| --- | --- |
| Twice yearly | 16 |
| Every 3-4 months | 15 |
| Monthly | 1 |
| Weekly | 1 |
| Total | 42 |

Additional comments on delirium education:

-Semi-helpful

-They are very informative

-Have scenarios where patients tested negative but was actually positive for delirium

-I think nurses often know how to identify delirium but doctors often don’t do any of the interventions to help prevent it.

-Please keep the current delirium tool – it’s the best one by far

Q10. During these past several months, when nurses on your unit recorded a positive delirium screen in MiChart, this would send alerts (pager and Best Practice Alert) to the covering physician, NP, or PA. Were you aware of this?

| Yes | 28 |
| --- | --- |
| No | 15 |
| Total | 43 |

Q11. If yes, how much did this information (i.e., that a pager alert and Best Practice Alert would be sent to the covering clinician) motive you to screen for delirium?


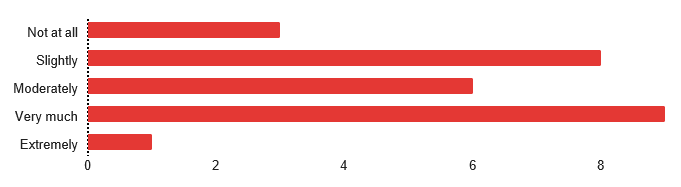


| Not at all | 3 |
| --- | --- |
| Slightly | 8 |
| Moderately | 6 |
| Very much | 9 |
| Extremely | 1 |
| Total | 27 |

Q12. If yes, how much did this information (i.e., that a pager alert and Best Practice Alert would be sent to the covering clinician) motive you to record positive delirium screens?


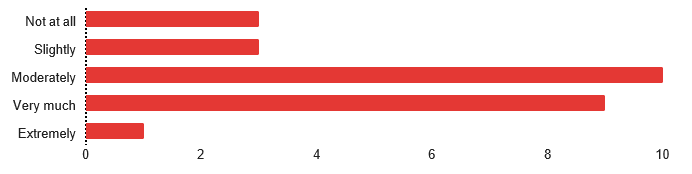


| Not at all | 3 |
| --- | --- |
| Slightly | 3 |
| Moderately | 10 |
| Very much | 9 |
| Extremely | 1 |
| Total | 26 |

Q13. Moving forward, how much would this information (i.e., that a pager alert and/or Best Practice Alert would be sent to the covering clinician with a positive delirium nursing screen) motivate you to screen for delirium?


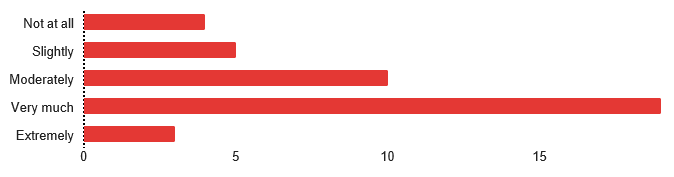


| Not at all | 4 |
| --- | --- |
| Slightly | 5 |
| Moderately | 10 |
| Very much | 19 |
| Extremely | 3 |
| Total | 41 |

Q14. Moving forward, how much would this information (i.e., that a pager alert and/or Best Practice Alert would be sent to the covering clinician with a positive delirium nursing screen) motivate you to record positive delirium screens?


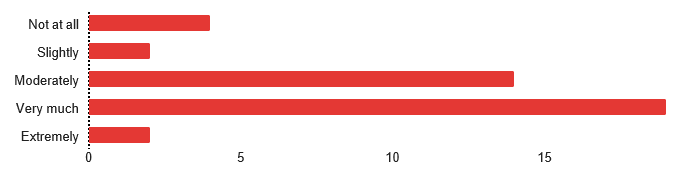


| Not at all | 4 |
| --- | --- |
| Slightly | 2 |
| Moderately | 14 |
| Very much | 19 |
| Extremely | 2 |
| Total | 41 |

Q14. Was the 4AT time-consuming?


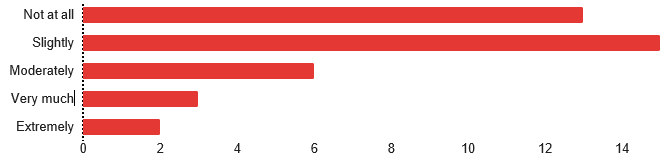


| Not at all | 13 |
| --- | --- |
| Slightly | 15 |
| Moderately | 6 |
| Very much | 3 |
| Extremely | 2 |
| Total | 39 |

Q15. Was the NuDesc time-consuming?


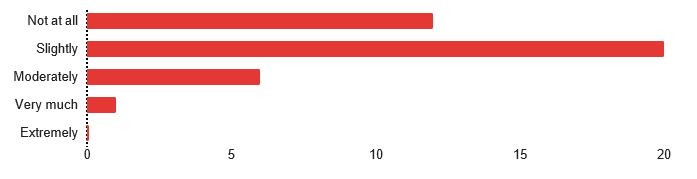


| Not at all | 12 |
| --- | --- |
| Slightly | 20 |
| Moderately | 6 |
| Very much | 1 |
| Extremely | 0 |
| Total | 39 |

Q16. Was the CAM time-consuming?


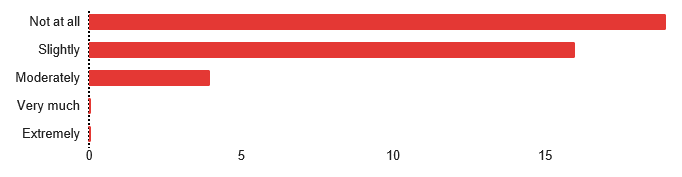


| Not at all | 19 |
| --- | --- |
| Slightly | 16 |
| Moderately | 4 |
| Very much | 0 |
| Extremely | 0 |
| Total | 39 |

Q17. How user-friendly was the 4AT?


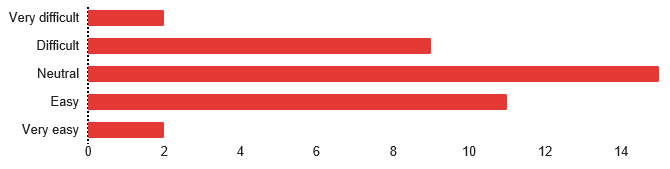


| Very difficult | 2 |
| --- | --- |
| Difficult | 9 |
| Neutral | 15 |
| Easy | 11 |
| Very easy | 2 |
| Total | 39 |

Q18. How user-friendly was the NuDesc?


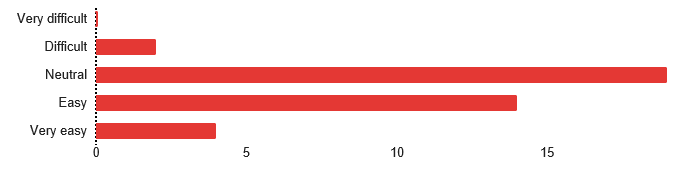


| Very difficult | 0 |
| --- | --- |
| Difficult | 2 |
| Neutral | 19 |
| Easy | 14 |
| Very easy | 4 |
| Total | 39 |

Q19. How user-friendly was the CAM?


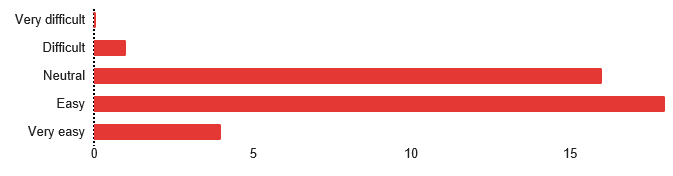


| Very difficult | 0 |
| --- | --- |
| Difficult | 1 |
| Neutral | 16 |
| Easy | 18 |
| Very easy | 4 |
| Total | 39 |

Q20. In your view, how accurate was the 4AT with identifying delirium?


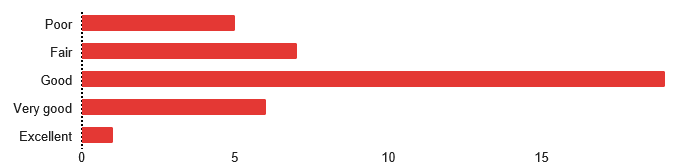


| Poor | 5 |
| --- | --- |
| Fair | 7 |
| Good | 19 |
| Very good | 6 |
| Excellent | 1 |
| Total | 38 |

Q21. In your view, how accurate was the NuDesc with identifying delirium?


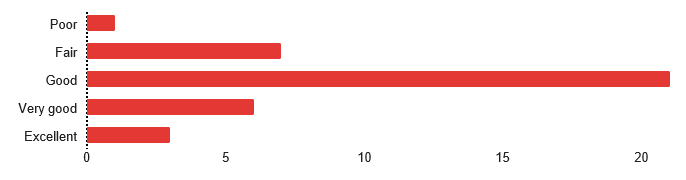


| Poor | 1 |
| --- | --- |
| Fair | 7 |
| Good | 21 |
| Very good | 6 |
| Excellent | 3 |
| Total | 38 |

Q21. In your view, how accurate was the CAM with identifying delirium?


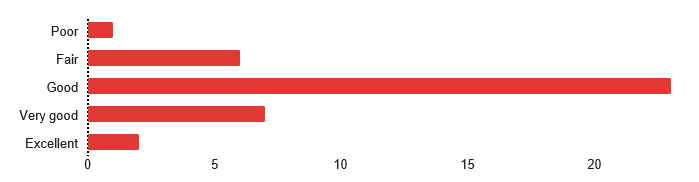


| Poor | 1 |
| --- | --- |
| Fair | 6 |
| Good | 23 |
| Very good | 7 |
| Excellent | 2 |
| Total | 39 |

Q22. What is your comfort level with the 4AT?


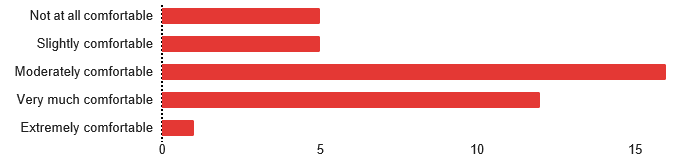


| Not at all comfortable | 5 |
| --- | --- |
| Slightly comfortable | 5 |
| Moderately comfortable | 16 |
| Very much comfortable | 12 |
| Extremely comfortable | 1 |
| Total | 39 |

Q23. What is your comfort level with the NuDesc?


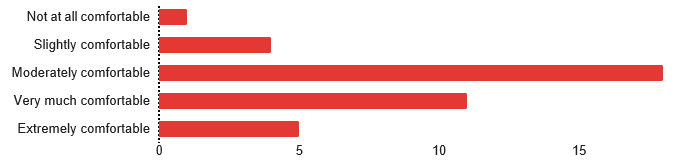


| Not at all comfortable | 1 |
| --- | --- |
| Slightly comfortable | 4 |
| Moderately comfortable | 18 |
| Very much comfortable | 11 |
| Extremely comfortable | 5 |
| Total | 39 |

Q24. What is your comfort level with the CAM?


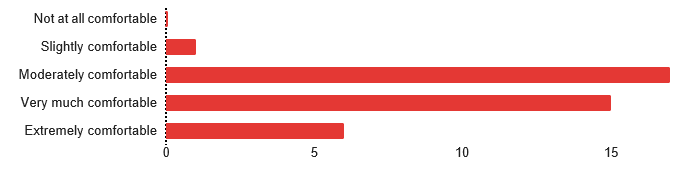


| Not at all comfortable | 0 |
| --- | --- |
| Slightly comfortable | 1 |
| Moderately comfortable | 17 |
| Very much comfortable | 15 |
| Extremely comfortable | 6 |
| Total | 39 |

Q25. Please rank in order your preferred delirium screening tool (1 – most preferred, 2 – second most preferred, 3 – last preferred):


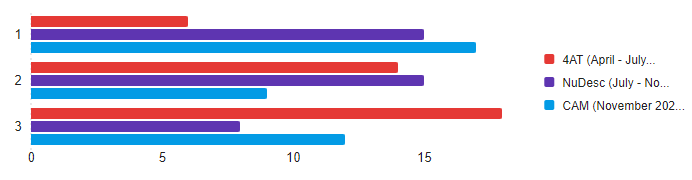


| Instrument | Top Choice | Second Choice | Third Choice | Total |
| --- | --- | --- | --- | --- |
| 4AT (April - July 2023) | 6 | 14 | 18 | 38 |
| NuDesc (July - November 2023) | 15 | 15 | 8 | 38 |
| CAM (November 2023 - February 2024) | 17 | 9 | 12 | 38 |

Please share any additional information that would be useful pertaining to delirium screening and management:

When a positive delirium screening is present having a conversation that consists of the bedside nurse, pharmacy, and primary service about interventions to address the delirium and following up with family. This does not happen often as it should.

N/A

Probably best not to change them in short sequence, I ask screening questions on autopilot and had to keep going back in rooms to ask the different delirium questions because I would forget that they had changed. Having them only change for some of the population did not help with this; it would be better to do all or none.

Pts can be forgetful or Aox4 and still unable to use the 4AT, the NuDesc was easier to use because the information is built into your assessment, and I think offers more detail that can appropriately assess pts for delirium. the cam is very basic, I think delirious pts can sometimes slip through the cracks with the cam.

Q2 turns seem to increase sleep deprivation and increase risk of delirium.

# **Supplementary Table S4 – Cohort Characteristics**

Bivariable Comparisons

|  |  | **Positive Delirium Screens (n=152 hospitalizations)** | | **Negative Delirium Screens (n=524 hospitalizations)** | |  |
| --- | --- | --- | --- | --- | --- | --- |
| Baseline Characteristics | **Variable** | **Mean or Proportion** | **SD** | **Mean or Proportion** | **SD** | **p-value** |
|  | **Age (y)** | **82** | **7.5** | **78.20** | **6.20** | **<0.001** |
|  | Male sex | 41% | -- | 48% | -- | 0.088 |
|  | **Length of Stay (d)** | **13** | **11** | **7** | **11** | **<0.001** |
|  | **Adjusted length of stay (d)** | **3** | **2.4** | **2** | **5.9** | **0.038** |
|  | **Adjusted length of stay with non-DRG (d)** | **4.5** | **5.3** | **2.5** | **6.1** | **<0.001** |
|  | **WRVUs** | **66.34** | **56.83** | **48.40** | **47.39** | **<0.001** |
| Comorbidities | AIDS/HIV (%) | 0% | -- | 0% | -- | 0.591 |
|  | Cerebrovascular Disease (%) | 40% | -- | 34% | -- | 0.182 |
|  | Chronic Pulmonary Disease (%) | 35% | -- | 40% | -- | 0.239 |
|  | Congestive Heart Failure (%) | 38% | -- | 34% | -- | 0.307 |
|  | **Dementia (%)** | **44%** | -- | **9%** | -- | **<0.001** |
|  | Diabetes With Chronic Complication (%) | 28% | -- | 23% | -- | 0.137 |
|  | Diabetes Without Chronic Complications (%) | 34% | -- | 38% | -- | 0.486 |
|  | Hemiplegia Paraplegia (%) | 7% | -- | 8% | -- | 0.615 |
|  | Malignancy (%) | 45% | -- | 49% | -- | 0.364 |
|  | Metastatic Solid Tumor (%) | 41% | -- | 48% | -- | 0.162 |
|  | Mild Liver Disease (%) | 20% | -- | 22% | -- | 0.638 |
|  | Moderate Severe Liver Disease (%) | 7% | -- | 5% | -- | 0.431 |
|  | Myocardial Infarction (%) | 20% | -- | 20% | -- | 0.947 |
|  | Peptic Ulcer Disease (%) | 13% | -- | 10% | -- | 0.396 |
|  | Peripheral Vascular Disease (%) | 50% | -- | 42% | -- | 0.093 |
|  | **Renal Disease (%)** | **50%** | -- | **39%** | -- | **0.013** |
|  | Rheumatic Disease (%) | 15% | -- | 15% | -- | 0.956 |
|  | **Charlson Comorbidity Score (%)** | **7.70** | **4.67** | **9.92** | **6.52** | **<0.001** |
| Disposition | **Home or Self Care (%)** | **19%** |  | **49%** |  | **<0.001** |
|  | Home-Health Care Service (%) | 16% |  | 20% |  | 0.267 |
|  | **Subacute Rehabilitation/Skilled Nursing Facility (%)** | **46%** |  | **23%** |  | **<0.001** |
|  | Acute/Inpatient Rehabilitation Facility (%) | 1.3% |  | 1.5% |  | 0.852 |
|  | **Deceased (%)** | **6%** |  | **1.9%** |  | **0.008** |
|  | **Hospice/Home (%)** | **3.3%** |  | **1%** |  | **0.035** |
|  | Hospice/Medical Facility (%) | 2.7% |  | 1.3% |  | 0.264 |
|  | Short Term Hospital (%) | 0% |  | 0.6% |  | 0.350 |
|  | Psychiatric Hospital (%) | 0% |  | 0.4% |  | 0.446 |
|  | **Long Term Care (%)** | **3.3%** |  | **0.4%** |  | **0.002** |
|  | Intermediate Care Facility (%) | 1.3% |  | 0.2% |  | 0.066 |
|  | Federal Hospital (%) | 0% |  | 0.4% |  | 0.446 |
|  | Discharged to Other Facility (%) | 0% |  | 0.4% |  | 0.446 |
|  | Nursing Facility (%) | 0.7% |  | 0% |  | 0.063 |
|  | Short term with planned readmission (%) | 0% |  | 0.4% |  | 0.446 |

Data presented based on available case counts. Abbreviatiosn: SD, standard deviation; DRG, Diagnosis-Related Group; WRVU, Work-Relative Value Units; AIDS, Acquired Immunodeficiency Syndrome; HIV, Human Immunodeficiency Virus;

# **Supplementary Text S5 – Admissions Based on Diagnosis-Related Group**

The following tables provide a detailed breakdown of admissions based on Diagnosis-Related Group (DRG). The tables also provide comparisons between patients who screened positive for delirium and those who did not screen positive for delirium based on each admission DRG category. Of note, a single admission may have multiple, overlapping DRG admission codes. The top eight most common DRG categories are listed.

1. Orthopedic/bone DRGs (206 admissions)

| **DRG** | **DRG Title** | **Count** |
| --- | --- | --- |
| 481 | HIP AND FEMUR PROCEDURES EXCEPT MAJOR JOINT WITH CC | 22 |
| 472 | CERVICAL SPINAL FUSION WITH CC | 17 |
| 470 | MAJOR HIP AND KNEE JOINT REPLACEMENT OR REATTACHMENT OF LOWER EXTREMITY WITHOUT MCC | 16 |
| 522 | HIP REPLACEMENT WITH PRINCIPAL DIAGNOSIS OF HIP FRACTURE WITHOUT MCC | 13 |
| 460 | SPINAL FUSION EXCEPT CERVICAL WITHOUT MCC | 10 |
| 467 | REVISION OF HIP OR KNEE REPLACEMENT WITH CC | 10 |
| 454 | COMBINED ANTERIOR AND POSTERIOR SPINAL FUSION WITH CC | 9 |
| 519 | BACK AND NECK PROCEDURES EXCEPT SPINAL FUSION WITH CC | 9 |
| 552 | MEDICAL BACK PROBLEMS WITHOUT MCC | 9 |
| 468 | REVISION OF HIP OR KNEE REPLACEMENT WITHOUT CC/MCC | 6 |
| 480 | HIP AND FEMUR PROCEDURES EXCEPT MAJOR JOINT WITH MCC | 5 |
| 483 | MAJOR JOINT OR LIMB REATTACHMENT PROCEDURES OF UPPER EXTREMITIES | 5 |
| 493 | LOWER EXTREMITY AND HUMERUS PROCEDURES EXCEPT HIP, FOOT AND FEMUR WITH CC | 5 |
| 956 | LIMB REATTACHMENT, HIP AND FEMUR PROCEDURES FOR MULTIPLE SIGNIFICANT TRAUMA | 4 |
| 521 | HIP REPLACEMENT WITH PRINCIPAL DIAGNOSIS OF HIP FRACTURE WITH MCC | 3 |
| 457 | SPINAL FUSION EXCEPT CERVICAL WITH SPINAL CURVATURE, MALIGNANCY, INFECTION OR EXTENSIVE FUSIONS WITH CC | 3 |
| 464 | WOUND DEBRIDEMENT AND SKIN GRAFT EXCEPT HAND FOR MUSCULOSKELETAL AND CONNECTIVE TISSUE DISORDERS WITH CC | 3 |
| 455 | COMBINED ANTERIOR AND POSTERIOR SPINAL FUSION WITHOUT CC/MCC | 3 |
| 459 | SPINAL FUSION EXCEPT CERVICAL WITH MCC | 3 |
| 466 | REVISION OF HIP OR KNEE REPLACEMENT WITH MCC | 3 |
| 469 | MAJOR HIP AND KNEE JOINT REPLACEMENT OR REATTACHMENT OF LOWER EXTREMITY WITH MCC OR TOTAL ANKLE REPLACEMENT | 3 |
| 478 | BIOPSIES OF MUSCULOSKELETAL SYSTEM AND CONNECTIVE TISSUE WITH CC | 3 |
| 515 | OTHER MUSCULOSKELETAL SYSTEM AND CONNECTIVE TISSUE O.R. PROCEDURES WITH MCC | 3 |
| 520 | BACK AND NECK PROCEDURES EXCEPT SPINAL FUSION WITHOUT CC/MCC | 3 |
| 536 | FRACTURES OF HIP AND PELVIS WITHOUT MCC | 2 |
| 563 | FRACTURE, SPRAIN, STRAIN AND DISLOCATION EXCEPT FEMUR, HIP, PELVIS AND THIGH WITHOUT MCC | 2 |
| 28 | SPINAL PROCEDURES WITH MCC | 2 |
| 453 | COMBINED ANTERIOR AND POSTERIOR SPINAL FUSION WITH MCC | 2 |
| 516 | OTHER MUSCULOSKELETAL SYSTEM AND CONNECTIVE TISSUE O.R. PROCEDURES WITH CC | 2 |
| 542 | PATHOLOGICAL FRACTURES AND MUSCULOSKELETAL AND CONNECTIVE TISSUE MALIGNANCY WITH MCC | 1 |
| 534 | FRACTURES OF FEMUR WITHOUT MCC | 1 |
| 562 | FRACTURE, SPRAIN, STRAIN AND DISLOCATION EXCEPT FEMUR, HIP, PELVIS AND THIGH WITH MCC | 1 |
| 463 | WOUND DEBRIDEMENT AND SKIN GRAFT EXCEPT HAND FOR MUSCULOSKELETAL AND CONNECTIVE TISSUE DISORDERS WITH MCC | 1 |
| 465 | WOUND DEBRIDEMENT AND SKIN GRAFT EXCEPT HAND FOR MUSCULOSKELETAL AND CONNECTIVE TISSUE DISORDERS WITHOUT CC/MCC | 1 |
| 486 | KNEE PROCEDURES WITH PRINCIPAL DIAGNOSIS OF INFECTION WITH CC | 1 |
| 510 | SHOULDER, ELBOW OR FOREARM PROCEDURES, EXCEPT MAJOR JOINT PROCEDURES WITH MCC | 1 |
| 511 | SHOULDER, ELBOW OR FOREARM PROCEDURES, EXCEPT MAJOR JOINT PROCEDURES WITH CC | 1 |
| 471 | CERVICAL SPINAL FUSION WITH MCC | 1 |
| 475 | AMPUTATION FOR MUSCULOSKELETAL SYSTEM AND CONNECTIVE TISSUE DISORDERS WITH CC | 1 |
| 477 | BIOPSIES OF MUSCULOSKELETAL SYSTEM AND CONNECTIVE TISSUE WITH MCC | 1 |
| 479 | BIOPSIES OF MUSCULOSKELETAL SYSTEM AND CONNECTIVE TISSUE WITHOUT CC/MCC | 1 |
| 482 | HIP AND FEMUR PROCEDURES EXCEPT MAJOR JOINT WITHOUT CC/MCC | 1 |
| 492 | LOWER EXTREMITY AND HUMERUS PROCEDURES EXCEPT HIP, FOOT AND FEMUR WITH MCC | 1 |
| 494 | LOWER EXTREMITY AND HUMERUS PROCEDURES EXCEPT HIP, FOOT AND FEMUR WITHOUT CC/MCC | 1 |
| 517 | OTHER MUSCULOSKELETAL SYSTEM AND CONNECTIVE TISSUE O.R. PROCEDURES WITHOUT CC/MCC | 1 |
| 518 | BACK AND NECK PROCEDURES EXCEPT SPINAL FUSION WITH MCC OR DISC DEVICE OR NEUROSTIMULATOR | 1 |
| 551 | MEDICAL BACK PROBLEMS WITH MCC | 1 |
| 553 | BONE DISEASES AND ARTHROPATHIES WITH MCC | 1 |
| 554 | BONE DISEASES AND ARTHROPATHIES WITHOUT MCC | 1 |
| 556 | SIGNS AND SYMPTOMS OF MUSCULOSKELETAL SYSTEM AND CONNECTIVE TISSUE WITHOUT MCC | 1 |
| 560 | AFTERCARE, MUSCULOSKELETAL SYSTEM AND CONNECTIVE TISSUE WITH CC | 1 |
| 561 | AFTERCARE, MUSCULOSKELETAL SYSTEM AND CONNECTIVE TISSUE WITHOUT CC/MCC | 1 |
| 565 | OTHER MUSCULOSKELETAL SYSTEM AND CONNECTIVE TISSUE DIAGNOSES WITH CC | 1 |
| 616 | AMPUTATION OF LOWER LIMB FOR ENDOCRINE, NUTRITIONAL AND METABOLIC DISORDERS WITH MCC | 1 |
| 617 | AMPUTATION OF LOWER LIMB FOR ENDOCRINE, NUTRITIONAL AND METABOLIC DISORDERS WITH CC | 1 |

Key Finding:

- Positive delirium screen patients were less to be hospitalized for orthopedic reasons compared to patients who did not screen positive for delirium (16.8% vs 33.7%, p<0.001)

1. Trauma (56 admissions)

| **DRG** | **DRG Name** | **Count** |
| --- | --- | --- |
| 522 | HIP REPLACEMENT WITH PRINCIPAL DIAGNOSIS OF HIP FRACTURE WITHOUT MCC | 13 |
| 956 | LIMB REATTACHMENT, HIP AND FEMUR PROCEDURES FOR MULTIPLE SIGNIFICANT TRAUMA | 4 |
| 521 | HIP REPLACEMENT WITH PRINCIPAL DIAGNOSIS OF HIP FRACTURE WITH MCC | 3 |
| 536 | FRACTURES OF HIP AND PELVIS WITHOUT MCC | 2 |
| 563 | FRACTURE, SPRAIN, STRAIN AND DISLOCATION EXCEPT FEMUR, HIP, PELVIS AND THIGH WITHOUT MCC | 2 |
| 542 | PATHOLOGICAL FRACTURES AND MUSCULOSKELETAL AND CONNECTIVE TISSUE MALIGNANCY WITH MCC | 1 |
| 534 | FRACTURES OF FEMUR WITHOUT MCC | 1 |
| 562 | FRACTURE, SPRAIN, STRAIN AND DISLOCATION EXCEPT FEMUR, HIP, PELVIS AND THIGH WITH MCC | 1 |
| 908 | OTHER O.R. PROCEDURES FOR INJURIES WITH CC | 4 |
| 964 | OTHER MULTIPLE SIGNIFICANT TRAUMA WITH CC | 4 |
| 862 | POSTOPERATIVE AND POST-TRAUMATIC INFECTIONS WITH MCC | 3 |
| 935 | NON-EXTENSIVE BURNS | 3 |
| 85 | TRAUMATIC STUPOR AND COMA <1 HOUR WITH MCC | 2 |
| 184 | MAJOR CHEST TRAUMA WITH CC | 2 |
| 605 | TRAUMA TO THE SKIN, SUBCUTANEOUS TISSUE AND BREAST WITHOUT MCC | 1 |
| 863 | POSTOPERATIVE AND POST-TRAUMATIC INFECTIONS WITHOUT MCC | 1 |
| 904 | SKIN GRAFTS FOR INJURIES WITH CC/MCC | 1 |
| 928 | FULL THICKNESS BURN WITH SKIN GRAFT OR INHALATION INJURY WITH CC/MCC | 1 |
| 82 | TRAUMATIC STUPOR AND COMA >1 HOUR WITH MCC | 1 |
| 83 | TRAUMATIC STUPOR AND COMA >1 HOUR WITH CC | 1 |
| 86 | TRAUMATIC STUPOR AND COMA <1 HOUR WITH CC | 1 |
| 902 | WOUND DEBRIDEMENTS FOR INJURIES WITH CC | 1 |
| 909 | OTHER O.R. PROCEDURES FOR INJURIES WITHOUT CC/MCC | 1 |
| 958 | OTHER O.R. PROCEDURES FOR MULTIPLE SIGNIFICANT TRAUMA WITH CC | 1 |
| 963 | OTHER MULTIPLE SIGNIFICANT TRAUMA WITH MCC | 1 |

Key Finding:

- There was no difference between patients with positive delirium screens and no positive screens for trauma-based hospitalizations (9.1% vs. 7.7%, p=0.625).

1. Infections (113 admissions)

| **DRG** | **DRG Name** | **Count** |
| --- | --- | --- |
| 457 | SPINAL FUSION EXCEPT CERVICAL WITH SPINAL CURVATURE, MALIGNANCY, INFECTION OR EXTENSIVE FUSIONS WITH CC | 3 |
| 464 | WOUND DEBRIDEMENT AND SKIN GRAFT EXCEPT HAND FOR MUSCULOSKELETAL AND CONNECTIVE TISSUE DISORDERS WITH CC | 3 |
| 463 | WOUND DEBRIDEMENT AND SKIN GRAFT EXCEPT HAND FOR MUSCULOSKELETAL AND CONNECTIVE TISSUE DISORDERS WITH MCC | 1 |
| 465 | WOUND DEBRIDEMENT AND SKIN GRAFT EXCEPT HAND FOR MUSCULOSKELETAL AND CONNECTIVE TISSUE DISORDERS WITHOUT CC/MCC | 1 |
| 486 | KNEE PROCEDURES WITH PRINCIPAL DIAGNOSIS OF INFECTION WITH CC | 1 |
| 862 | POSTOPERATIVE AND POST-TRAUMATIC INFECTIONS WITH MCC | 3 |
| 605 | TRAUMA TO THE SKIN, SUBCUTANEOUS TISSUE AND BREAST WITHOUT MCC | 1 |
| 863 | POSTOPERATIVE AND POST-TRAUMATIC INFECTIONS WITHOUT MCC | 1 |
| 904 | SKIN GRAFTS FOR INJURIES WITH CC/MCC | 1 |
| 928 | FULL THICKNESS BURN WITH SKIN GRAFT OR INHALATION INJURY WITH CC/MCC | 1 |
| 871 | SEPTICEMIA OR SEVERE SEPSIS WITHOUT MV >96 HOURS WITH MCC | 17 |
| 853 | INFECTIOUS AND PARASITIC DISEASES WITH O.R. PROCEDURES WITH MCC | 12 |
| 872 | SEPTICEMIA OR SEVERE SEPSIS WITHOUT MV >96 HOURS WITHOUT MCC | 8 |
| 392 | ESOPHAGITIS, GASTROENTERITIS AND MISCELLANEOUS DIGESTIVE DISORDERS WITHOUT MCC | 6 |
| 177 | RESPIRATORY INFECTIONS AND INFLAMMATIONS WITH MCC | 6 |
| 690 | KIDNEY AND URINARY TRACT INFECTIONS WITHOUT MCC | 5 |
| 391 | ESOPHAGITIS, GASTROENTERITIS AND MISCELLANEOUS DIGESTIVE DISORDERS WITH MCC | 4 |
| 371 | MAJOR GASTROINTESTINAL DISORDERS AND PERITONEAL INFECTIONS WITH MCC | 3 |
| 193 | SIMPLE PNEUMONIA AND PLEURISY WITH MCC | 3 |
| 372 | MAJOR GASTROINTESTINAL DISORDERS AND PERITONEAL INFECTIONS WITH CC | 2 |
| 373 | MAJOR GASTROINTESTINAL DISORDERS AND PERITONEAL INFECTIONS WITHOUT CC/MCC | 2 |
| 689 | KIDNEY AND URINARY TRACT INFECTIONS WITH MCC | 2 |
| 502 | SOFT TISSUE PROCEDURES WITHOUT CC/MCC | 2 |
| 540 | OSTEOMYELITIS WITH CC | 2 |
| 602 | CELLULITIS WITH MCC | 2 |
| 603 | CELLULITIS WITHOUT MCC | 2 |
| 720 | SEPTICEMIA AND DISSEMINATED INFECTIONS | 2 |
| 870 | SEPTICEMIA OR SEVERE SEPSIS WITH MV >96 HOURS | 2 |
| 339 | APPENDECTOMY WITH COMPLICATED PRINCIPAL DIAGNOSIS WITH CC | 1 |
| 178 | RESPIRATORY INFECTIONS AND INFLAMMATIONS WITH CC | 1 |
| 179 | RESPIRATORY INFECTIONS AND INFLAMMATIONS WITHOUT CC/MCC | 1 |
| 152 | OTITIS MEDIA AND URI WITH MCC | 1 |
| 153 | OTITIS MEDIA AND URI WITHOUT MCC | 1 |
| 194 | SIMPLE PNEUMONIA AND PLEURISY WITH CC | 1 |
| 380 | SKIN ULCERS | 1 |
| 500 | SOFT TISSUE PROCEDURES WITH MCC | 1 |
| 539 | OSTEOMYELITIS WITH MCC | 1 |
| 596 | MAJOR SKIN DISORDERS WITHOUT MCC | 1 |
| 607 | MINOR SKIN DISORDERS WITHOUT MCC | 1 |
| 622 | SKIN GRAFTS AND WOUND DEBRIDEMENT FOR ENDOCRINE, NUTRITIONAL AND METABOLIC DISORDERS WITH MCC | 1 |
| 623 | SKIN GRAFTS AND WOUND DEBRIDEMENT FOR ENDOCRINE, NUTRITIONAL AND METABOLIC DISORDERS WITH CC | 1 |
| 854 | INFECTIOUS AND PARASITIC DISEASES WITH O.R. PROCEDURES WITH CC | 1 |
| 867 | OTHER INFECTIOUS AND PARASITIC DISEASES DIAGNOSES WITH MCC | 1 |

Key Finding:

- Delirium-positive patients were more likely to be admitted for infectious reasons compared to patients without a positive delirium screen (22.1% vs 14.8%, p=0.032)

1. Gastrointestinal tract diseases (104 admissions)

| **DRG** | **DRG Name** | **Count** |
| --- | --- | --- |
| 510 | SHOULDER, ELBOW OR FOREARM PROCEDURES, EXCEPT MAJOR JOINT PROCEDURES WITH MCC | 1 |
| 511 | SHOULDER, ELBOW OR FOREARM PROCEDURES, EXCEPT MAJOR JOINT PROCEDURES WITH CC | 1 |
| 392 | ESOPHAGITIS, GASTROENTERITIS AND MISCELLANEOUS DIGESTIVE DISORDERS WITHOUT MCC | 6 |
| 391 | ESOPHAGITIS, GASTROENTERITIS AND MISCELLANEOUS DIGESTIVE DISORDERS WITH MCC | 4 |
| 371 | MAJOR GASTROINTESTINAL DISORDERS AND PERITONEAL INFECTIONS WITH MCC | 3 |
| 372 | MAJOR GASTROINTESTINAL DISORDERS AND PERITONEAL INFECTIONS WITH CC | 2 |
| 373 | MAJOR GASTROINTESTINAL DISORDERS AND PERITONEAL INFECTIONS WITHOUT CC/MCC | 2 |
| 339 | APPENDECTOMY WITH COMPLICATED PRINCIPAL DIAGNOSIS WITH CC | 1 |
| 393 | OTHER DIGESTIVE SYSTEM DIAGNOSES WITH MCC | 7 |
| 374 | DIGESTIVE MALIGNANCY WITH MCC | 6 |
| 394 | OTHER DIGESTIVE SYSTEM DIAGNOSES WITH CC | 6 |
| 329 | MAJOR SMALL AND LARGE BOWEL PROCEDURES WITH MCC | 5 |
| 378 | GASTROINTESTINAL HEMORRHAGE WITH CC | 5 |
| 375 | DIGESTIVE MALIGNANCY WITH CC | 4 |
| 441 | DISORDERS OF LIVER EXCEPT MALIGNANCY, CIRRHOSIS OR ALCOHOLIC HEPATITIS WITH MCC | 3 |
| 377 | GASTROINTESTINAL HEMORRHAGE WITH MCC | 3 |
| 389 | GASTROINTESTINAL OBSTRUCTION WITH CC | 3 |
| 390 | GASTROINTESTINAL OBSTRUCTION WITHOUT CC/MCC | 3 |
| 884 | ORGANIC DISTURBANCES AND INTELLECTUAL DISABILITY | 3 |
| 439 | DISORDERS OF PANCREAS EXCEPT MALIGNANCY WITH CC | 2 |
| 442 | DISORDERS OF LIVER EXCEPT MALIGNANCY, CIRRHOSIS OR ALCOHOLIC HEPATITIS WITH CC | 2 |
| 327 | STOMACH, ESOPHAGEAL AND DUODENAL PROCEDURES WITH CC | 2 |
| 330 | MAJOR SMALL AND LARGE BOWEL PROCEDURES WITH CC | 2 |
| 335 | PERITONEAL ADHESIOLYSIS WITH MCC | 2 |
| 336 | PERITONEAL ADHESIOLYSIS WITH CC | 2 |
| 357 | OTHER DIGESTIVE SYSTEM O.R. PROCEDURES WITH CC | 2 |
| 379 | GASTROINTESTINAL HEMORRHAGE WITHOUT CC/MCC | 2 |
| 388 | GASTROINTESTINAL OBSTRUCTION WITH MCC | 2 |
| 406 | PANCREAS, LIVER AND SHUNT PROCEDURES WITH CC | 2 |
| 444 | DISORDERS OF THE BILIARY TRACT WITH MCC | 2 |
| 436 | MALIGNANCY OF HEPATOBILIARY SYSTEM OR PANCREAS WITH CC | 1 |
| 5 | LIVER TRANSPLANT WITH MCC OR INTESTINAL TRANSPLANT | 1 |
| 326 | STOMACH, ESOPHAGEAL AND DUODENAL PROCEDURES WITH MCC | 1 |
| 328 | STOMACH, ESOPHAGEAL AND DUODENAL PROCEDURES WITHOUT CC/MCC | 1 |
| 344 | MINOR SMALL AND LARGE BOWEL PROCEDURES WITH MCC | 1 |
| 348 | ANAL AND STOMAL PROCEDURES WITH CC | 1 |
| 368 | MAJOR ESOPHAGEAL DISORDERS WITH MCC | 1 |
| 395 | OTHER DIGESTIVE SYSTEM DIAGNOSES WITHOUT CC/MCC | 1 |
| 409 | BILIARY TRACT PROCEDURES EXCEPT ONLY CHOLECYSTECTOMY WITH OR WITHOUT C.D.E. WITH CC | 1 |
| 414 | CHOLECYSTECTOMY EXCEPT BY LAPAROSCOPE WITHOUT C.D.E. WITH MCC | 1 |
| 419 | LAPAROSCOPIC CHOLECYSTECTOMY WITHOUT C.D.E. WITHOUT CC/MCC | 1 |
| 432 | CIRRHOSIS AND ALCOHOLIC HEPATITIS WITH MCC | 1 |
| 445 | DISORDERS OF THE BILIARY TRACT WITH CC | 1 |
| 446 | DISORDERS OF THE BILIARY TRACT WITHOUT CC/MCC | 1 |

Key finding:

- Patients who screened positive for delirium were less likely to have gastrointestinal reason for admission compared to those without a positive delirium screen (6.50% vs 17.6%, p<0.001)

5. Hematologic/Oncologic (80 Admissions)

| **DRG** | **DRG Name** | **Count** |
| --- | --- | --- |
| 457 | SPINAL FUSION EXCEPT CERVICAL WITH SPINAL CURVATURE, MALIGNANCY, INFECTION OR EXTENSIVE FUSIONS WITH CC | 3 |
| 542 | PATHOLOGICAL FRACTURES AND MUSCULOSKELETAL AND CONNECTIVE TISSUE MALIGNANCY WITH MCC | 1 |
| 605 | TRAUMA TO THE SKIN, SUBCUTANEOUS TISSUE AND BREAST WITHOUT MCC | 1 |
| 392 | ESOPHAGITIS, GASTROENTERITIS AND MISCELLANEOUS DIGESTIVE DISORDERS WITHOUT MCC | 6 |
| 391 | ESOPHAGITIS, GASTROENTERITIS AND MISCELLANEOUS DIGESTIVE DISORDERS WITH MCC | 4 |
| 374 | DIGESTIVE MALIGNANCY WITH MCC | 6 |
| 375 | DIGESTIVE MALIGNANCY WITH CC | 4 |
| 441 | DISORDERS OF LIVER EXCEPT MALIGNANCY, CIRRHOSIS OR ALCOHOLIC HEPATITIS WITH MCC | 3 |
| 439 | DISORDERS OF PANCREAS EXCEPT MALIGNANCY WITH CC | 2 |
| 442 | DISORDERS OF LIVER EXCEPT MALIGNANCY, CIRRHOSIS OR ALCOHOLIC HEPATITIS WITH CC | 2 |
| 436 | MALIGNANCY OF HEPATOBILIARY SYSTEM OR PANCREAS WITH CC | 1 |
| 640 | MISCELLANEOUS DISORDERS OF NUTRITION, METABOLISM, FLUIDS AND ELECTROLYTES WITH MCC | 8 |
| 641 | MISCELLANEOUS DISORDERS OF NUTRITION, METABOLISM, FLUIDS AND ELECTROLYTES WITHOUT MCC | 6 |
| 657 | KIDNEY AND URETER PROCEDURES FOR NEOPLASM WITH CC | 5 |
| 181 | RESPIRATORY NEOPLASMS WITH CC | 3 |
| 812 | RED BLOOD CELL DISORDERS WITHOUT MCC | 3 |
| 598 | MALIGNANT BREAST DISORDERS WITH CC | 2 |
| 739 | UTERINE AND ADNEXA PROCEDURES FOR NON-OVARIAN AND NON-ADNEXAL MALIGNANCY WITH MCC | 2 |
| 840 | LYMPHOMA AND NON-ACUTE LEUKEMIA WITH MCC | 2 |
| 180 | RESPIRATORY NEOPLASMS WITH MCC | 1 |
| 246 | PERCUTANEOUS CARDIOVASCULAR PROCEDURES WITH DRUG-ELUTING STENT WITH MCC OR 4+ ARTERIES OR STENTS | 1 |
| 656 | KIDNEY AND URETER PROCEDURES FOR NEOPLASM WITH MCC | 1 |
| 658 | KIDNEY AND URETER PROCEDURES FOR NEOPLASM WITHOUT CC/MCC | 1 |
| 659 | KIDNEY AND URETER PROCEDURES FOR NON-NEOPLASM WITH MCC | 1 |
| 686 | KIDNEY AND URINARY TRACT NEOPLASMS WITH MCC | 1 |
| 687 | KIDNEY AND URINARY TRACT NEOPLASMS WITH CC | 1 |
| 54 | NERVOUS SYSTEM NEOPLASMS WITH MCC | 1 |
| 722 | MALIGNANCY, MALE REPRODUCTIVE SYSTEM WITH MCC | 1 |
| 737 | UTERINE AND ADNEXA PROCEDURES FOR OVARIAN OR ADNEXAL MALIGNANCY WITH CC | 1 |
| 803 | OTHER O.R. PROCEDURES OF THE BLOOD AND BLOOD FORMING ORGANS WITH CC | 1 |
| 809 | MAJOR HEMATOLOGICAL AND IMMUNOLOGICAL DIAGNOSES EXCEPT SICKLE CELL CRISIS AND COAGULATION DISORDERS WITH CC | 1 |
| 811 | RED BLOOD CELL DISORDERS WITH MCC | 1 |
| 821 | LYMPHOMA AND LEUKEMIA WITH MAJOR O.R. PROCEDURES WITH CC | 1 |
| 834 | ACUTE LEUKEMIA WITHOUT MAJOR O.R. PROCEDURES WITH MCC | 1 |
| 841 | LYMPHOMA AND NON-ACUTE LEUKEMIA WITH CC | 1 |

- The proportion of patients with positive delirium screens was similar to non-delirious patients for being hospitalized for hematology/oncology reasons (9.7% vs 12.2%, p=0.408)

1. Cardiovascular/peripheral vascular/lung (54 admissions)

| **DRG** | **DRG Name** | **Count** |
| --- | --- | --- |
| 177 | RESPIRATORY INFECTIONS AND INFLAMMATIONS WITH MCC | 6 |
| 178 | RESPIRATORY INFECTIONS AND INFLAMMATIONS WITH CC | 1 |
| 179 | RESPIRATORY INFECTIONS AND INFLAMMATIONS WITHOUT CC/MCC | 1 |
| 181 | RESPIRATORY NEOPLASMS WITH CC | 3 |
| 180 | RESPIRATORY NEOPLASMS WITH MCC | 1 |
| 246 | PERCUTANEOUS CARDIOVASCULAR PROCEDURES WITH DRUG-ELUTING STENT WITH MCC OR 4+ ARTERIES OR STENTS | 1 |
| 189 | PULMONARY EDEMA AND RESPIRATORY FAILURE | 8 |
| 291 | HEART FAILURE AND SHOCK WITH MCC | 5 |
| 175 | PULMONARY EMBOLISM WITH MCC OR ACUTE COR PULMONALE | 4 |
| 190 | CHRONIC OBSTRUCTIVE PULMONARY DISEASE WITH MCC | 2 |
| 196 | INTERSTITIAL LUNG DISEASE WITH MCC | 2 |
| 270 | OTHER MAJOR CARDIOVASCULAR PROCEDURES WITH MCC | 2 |
| 300 | PERIPHERAL VASCULAR DISORDERS WITH CC | 2 |
| 314 | OTHER CIRCULATORY SYSTEM DIAGNOSES WITH MCC | 2 |
| 20 | INTRACRANIAL VASCULAR PROCEDURES WITH PRINCIPAL DIAGNOSIS HEMORRHAGE WITH MCC | 1 |
| 70 | NONSPECIFIC CEREBROVASCULAR DISORDERS WITH MCC | 1 |
| 268 | AORTIC AND HEART ASSIST PROCEDURES EXCEPT PULSATION BALLOON WITH MCC | 1 |
| 176 | PULMONARY EMBOLISM WITHOUT MCC | 1 |
| 204 | RESPIRATORY SIGNS AND SYMPTOMS | 1 |
| 205 | OTHER RESPIRATORY SYSTEM DIAGNOSES WITH MCC | 1 |
| 206 | OTHER RESPIRATORY SYSTEM DIAGNOSES WITHOUT MCC | 1 |
| 207 | RESPIRATORY SYSTEM DIAGNOSIS WITH VENTILATOR SUPPORT >96 HOURS | 1 |
| 260 | CARDIAC PACEMAKER REVISION EXCEPT DEVICE REPLACEMENT WITH MCC | 1 |
| 267 | ENDOVASCULAR CARDIAC VALVE REPLACEMENT AND SUPPLEMENT PROCEDURES WITHOUT MCC | 1 |
| 280 | ACUTE MYOCARDIAL INFARCTION, DISCHARGED ALIVE WITH MCC | 1 |
| 281 | ACUTE MYOCARDIAL INFARCTION, DISCHARGED ALIVE WITH CC | 1 |
| 286 | CIRCULATORY DISORDERS EXCEPT AMI, WITH CARDIAC CATHETERIZATION WITH MCC | 1 |
| 301 | PERIPHERAL VASCULAR DISORDERS WITHOUT CC/MCC | 1 |

Key Finding:

- Admission rates were similar between delirium-positive and delirium-negative patients (7.8% vs. 7.9%, p=0.976)

1. Renal (38 admissions)

| **DRG** | **DRG Name** | **Count** |
| --- | --- | --- |
| 690 | KIDNEY AND URINARY TRACT INFECTIONS WITHOUT MCC | 5 |
| 689 | KIDNEY AND URINARY TRACT INFECTIONS WITH MCC | 2 |
| 657 | KIDNEY AND URETER PROCEDURES FOR NEOPLASM WITH CC | 5 |
| 656 | KIDNEY AND URETER PROCEDURES FOR NEOPLASM WITH MCC | 1 |
| 658 | KIDNEY AND URETER PROCEDURES FOR NEOPLASM WITHOUT CC/MCC | 1 |
| 659 | KIDNEY AND URETER PROCEDURES FOR NON-NEOPLASM WITH MCC | 1 |
| 686 | KIDNEY AND URINARY TRACT NEOPLASMS WITH MCC | 1 |
| 687 | KIDNEY AND URINARY TRACT NEOPLASMS WITH CC | 1 |
| 698 | OTHER KIDNEY AND URINARY TRACT DIAGNOSES WITH MCC | 7 |
| 683 | RENAL FAILURE WITH CC | 6 |
| 682 | RENAL FAILURE WITH MCC | 5 |
| 699 | OTHER KIDNEY AND URINARY TRACT DIAGNOSES WITH CC | 2 |
| 696 | KIDNEY AND URINARY TRACT SIGNS AND SYMPTOMS WITHOUT MCC | 1 |

Key Finding:

- Admission rates were similar between delirium-positive and delirium-negative patients (4.5% vs. 5.8%, p=0.547)

1. Other neurologic conditions (21 admissions)

| DRG | DRG Name | Count |
| --- | --- | --- |
| 54 | NERVOUS SYSTEM NEOPLASMS WITH MCC | 1 |
| 20 | INTRACRANIAL VASCULAR PROCEDURES WITH PRINCIPAL DIAGNOSIS HEMORRHAGE WITH MCC | 1 |
| 70 | NONSPECIFIC CEREBROVASCULAR DISORDERS WITH MCC | 1 |
| 92 | OTHER DISORDERS OF NERVOUS SYSTEM WITH CC | 5 |
| 57 | DEGENERATIVE NERVOUS SYSTEM DISORDERS WITHOUT MCC | 4 |
| 64 | INTRACRANIAL HEMORRHAGE OR CEREBRAL INFARCTION WITH MCC | 2 |
| 91 | OTHER DISORDERS OF NERVOUS SYSTEM WITH MCC | 2 |
| 31 | VENTRICULAR SHUNT PROCEDURES WITH MCC | 1 |
| 40 | PERIPHERAL, CRANIAL NERVE AND OTHER NERVOUS SYSTEM PROCEDURES WITH MCC | 1 |
| 56 | DEGENERATIVE NERVOUS SYSTEM DISORDERS WITH MCC | 1 |
| 100 | SEIZURES WITH MCC | 1 |
| 101 | SEIZURES WITHOUT MCC | 1 |

Key finding:

- Patients with a positive delirium screen were more likely to be admitted for neurologic conditions compared to patients without a positive delirium screen (6.5% vs 2.1%, p=0.005)
